# Comparing blood pressure measurements between a photoplethysmography-based and a standard cuff-based manometry device

**DOI:** 10.1038/s41598-020-73172-3

**Published:** 2020-09-30

**Authors:** Dean Nachman, Yftach Gepner, Nir Goldstein, Eli Kabakov, Arik Ben Ishay, Romi Littman, Yuval Azmon, Eli Jaffe, Arik Eisenkraft

**Affiliations:** 1 Institute for Research in Military Medicine, Faculty of Medicine, The Hebrew University of Jerusalem and the Israel Defense Force Medical Corps, POB 12272, 91120 Jerusalem, Israel; 2 Heart Institute, Hadassah Ein Kerem Medical Center, Jerusalem, Israel; 3 Department of Epidemiology and Preventive Medicine, School of Public Health, Sackler Faculty of Medicine, and Sylvan Adams Sports Institute, Tel-Aviv University, Tel-Aviv, Israel; 4 Biobeat Technologies LTD, Petah Tikva, Israel; 5 Obstetrics and Gynecology Department, Hillel Yaffe Medical Center and The Rappaport Faculty of Medicine, The Technion, Haifa, Israel; 6 Magen David Adom, Israel National Emergency Medical Services, Kiryat Ono, Israel

**Keywords:** Physiology, Cardiology, Health care

## Abstract

Repeated blood pressure (BP) measurements allow better control of hypertension. Current measurements rely on cuff-based devices. The aim of the present study was to compare BP measurements using a novel cuff-less photoplethysmography-based device to a standard sphygmomanometer device. Males and females were recruited from within the general population who arrived at a public BP screening station. One to two measurements were taken from each using a sphygmomanometer-based and the photoplethysmography-based devices. Devices were considered equal if the mean difference between paired measurements was below 5 mmHg and the Standard Deviation (SD) was no greater than 8 mmHg. Agreement and reliability analyses were also performed. 1057 subjects were included in the study analysis. There were no adverse events during the study. The mean (± SD) difference between paired measurements for all subjects was -0.1 ± 3.6 mmHg for the systolic and 0.0 ± 3.5 mmHg for the diastolic readings. We found 96.31% agreement in identifying hypertension and an Interclass Correlation Coefficient of 0.99 and 0.97 for systolic and diastolic measurements, respectively. The photoplethysmography-based device was found similar to the gold-standard sphygmomanometer-based device with high agreement and reliability levels. The device might enable a reliable, more convenient method for repeated BP monitoring.

## Introduction

Blood pressure (BP) is one of the basic measurements taken to evaluate the physiological status of an individual, whether in the pre-hospital setting, in a community healthcare clinic or in the hospital^1^. For over half a century it has been known that there is a strong relationship between abnormal blood pressure levels and the risk for health complications and death^2^. Higher levels of systolic and diastolic blood pressure have been associated with increased cardiovascular disease risk^2,3^. Currently used BP devices are usually based on the manometry method. This method is cumbersome, not always accurate and may have inaccurate measurements for different reasons, e.g. varying cuff size, accepted measurement guidelines not being followed, lack of routine calibration, and more^4–9^. There is a need to train both the operator and the patient on how to use these devices to allow valid measurements^10^. Despite these difficulties, and since hypertension is regarded as the world’s most common and modifiable cardiovascular risk factor^11,12^, BP measurements are still required in routine healthcare, with treatment often depending on imprecise measurements. Moreover, the new guideline issued by the American College of Cardiology and the American Heart Association (ACC/AHA) provides a substantially more stringent recommendation concerning out-of-office and self-monitored BP measurements, and recommends a more modern approach to out-of-office BP measurements using ambulatory or home BP monitoring to both confirm the diagnosis of hypertension and to titrate BP-lowering medications^12–15^.

Thus, there is a need for simple, non-invasive, wireless monitoring systems that will enable health systems to implement future strategies, allowing a dramatic change in the way we gather physiological data, with an emphasis on BP^16^. This will also allow proper home care monitoring as well as pre-hospital and in-hospital care, providing early identification and prevention of cardiovascular morbidity.

**Figure 1:**
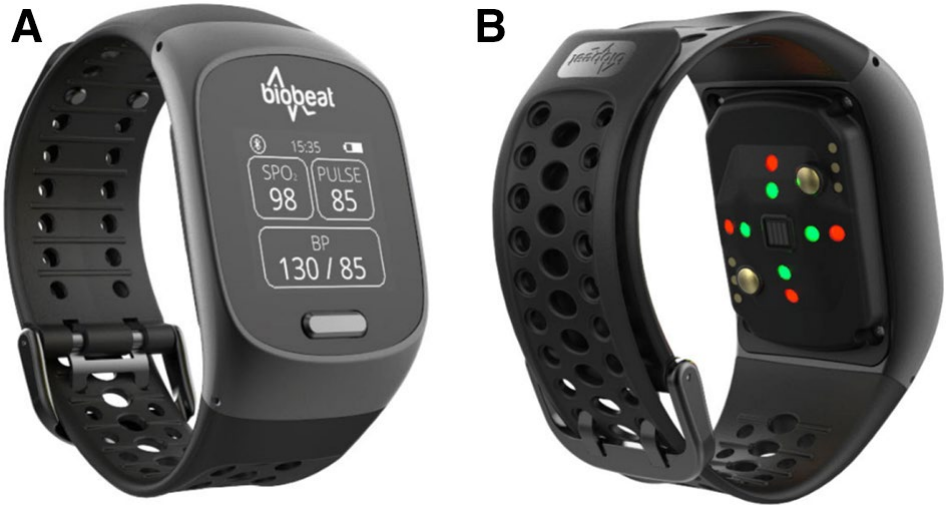
The Biobeat BB-613WP device. This PPG-based wearable provides non-invasive, cuffless, wireless and repeated measurement of blood pressure, heart rate, saturation, respiratory rate, stroke volume, cardiac output, cardiac index, systemic vascular resistance, pulse pressure, heart rate variability, mean arterial pressure, skin temperature, sweat, movement, and calories. Data is transmitted in real-time to a user App and to a medical management system. (**A**) The face side. (**B**) The back side with integrated sensors.

The aim of the current study was to compare non-invasive BP measurements between a commonly used manometry device and a new reflective photoplethysmography (PPG) device (BB-613WP, Biobeat Technologies LTD, Petah Tikva, Israel, Fig. 1). The wireless device is FDA cleared for spot check measurements of noninvasive cuffless blood pressure, saturation, and heart rate and CE cleared for several other vitals including stroke volume and cardiac output.

## Results

### Patient characteristics

Out of 1480 subjects recruited, 1057 subjects were included in the final analysis (466 males (44.1%) and 591 females (55.9%)), for which the defined data set was available (Table 1). The age range for recruited subjects was 8–100 years. 97 subjects (43 males (44.3%) and 54 females (55.7%)) reported a prior diagnosis of hypertension, 88 of them were under anti-hypertensive treatment. In 491 subjects a second BP measurement was taken after a short period of physical activity (5 min of strenuous walking). No adverse events were reported during the study.

**Table 1:** Characteristics, co-morbidities and habits of all 1057 participants.

Characteristic		
Age (years)	Mean ± SD	35.1 ± 23.8
Female Gender	N (%)	591 (55.9%)
Height (m)	Mean ± SD	1.67 ± 1
Weight (Kg)	Mean ± SD	68 ± 16
BMI (kg/m2)	Mean ± SD	24.1 ± 4.7
**Co-morbidities & habits**		
Hypertension	N (%)	97 (9.1%)
Smoking	N (%)	91 (8.6%)
Hypercholesterolemia	N (%)	66 (6.2%)
Diabetes	N (%)	52 (4.9%)
Asthma	N (%)	18 (1.7%)
IHD	N (%)	13 (1.2%)
Alcohol use	N (%)	11 (1%)
CHF	N (%)	10 (0.9%)
Other	N (%)	24 (2.3%)
Anemia	N (%)	8 (0.8%)
CVA	N (%)	5 (0.5%)
Cardiac arrhythmia	N (%)	4 (0.4%)
COPD	N (%)	4 (0.4%)
Migraines	N (%)	3 (0.3%)

All co-morbidities were diagnosed prior to participation in the study.

*SD* standard deviation, *BMI* body mass index, *N* number of participants. *IHD* ischemic heart disease. *CHF* congestive heart failure. *CVA* cerebrovascular accident, *COPD* chronic obstructive pulmonary disease.

### Similarity between the measurements of the devices

Table 2 presents the mean and SD measurements of the reference and the BB-613WP devices. The mean ± SD Systolic Blood Pressure (SBP) of the reference device was 117.1 ± 19.5 mmHg and with the BB-613WP 117.2 ± 19.9 mmHg (*p* < 0.001), the mean ± SD Diastolic Blood Pressure (DBP) of the reference device was 68.2 ± 18.7 mmHg and with the BB-613WP 68.2 ± 11.5 mmHg (*p* < 0.001). Reliability of the BB-613WP device with regards to the reference sphygmomanometer device was excellent for both systolic and diastolic measurements and in both the first and second measurements, as indicated by an ICC of 0.96 and above (Table 3).

**Table 2:** Comparison of systolic and diastolic pressures measured by both devices.

	Reference	BB-613WP	*P* value
**Systolic BP (mmHg)**			
N	1057	1057	< .001
Mean ± SD	117.1 ± 19.5	117.2 ± 19.9	
Minimum	70	71	
Maximum	220	223	
**Diastolic BP (mmHg)**			
N	1057	1057	< .001
Mean ± SD	68.2 ± 11.1	68.2 ± 11.5	
Minimum	40	41	
Maximum	110	112	

*BP* blood pressure, *N* number of participants.

**Table 3:** Reliability of the BB-613WP device with regards to the reference sphygmomanometer device as reflected in Intraclass Correlation Coefficient. Sys1—first systolic measurement; Sys2—second systolic measurement; Dias1—first diastolic measurement; Dias2—second diastolic measurement.

	Sys1	Sys2	Dias1	Dias2
Value	0.99	0.98	0.98	0.96
**Intraclass Correlation Coefficient**				
* 95%CI*				
Upper limit	0.99	0.99	0.97	0.95
Lower Limit	0.99	0.99	0.98	0.97
*P* value	< .001	< .001	< .001	< .001

### Evaluation of the delta between the measurements in both devices

**Figure 2:**
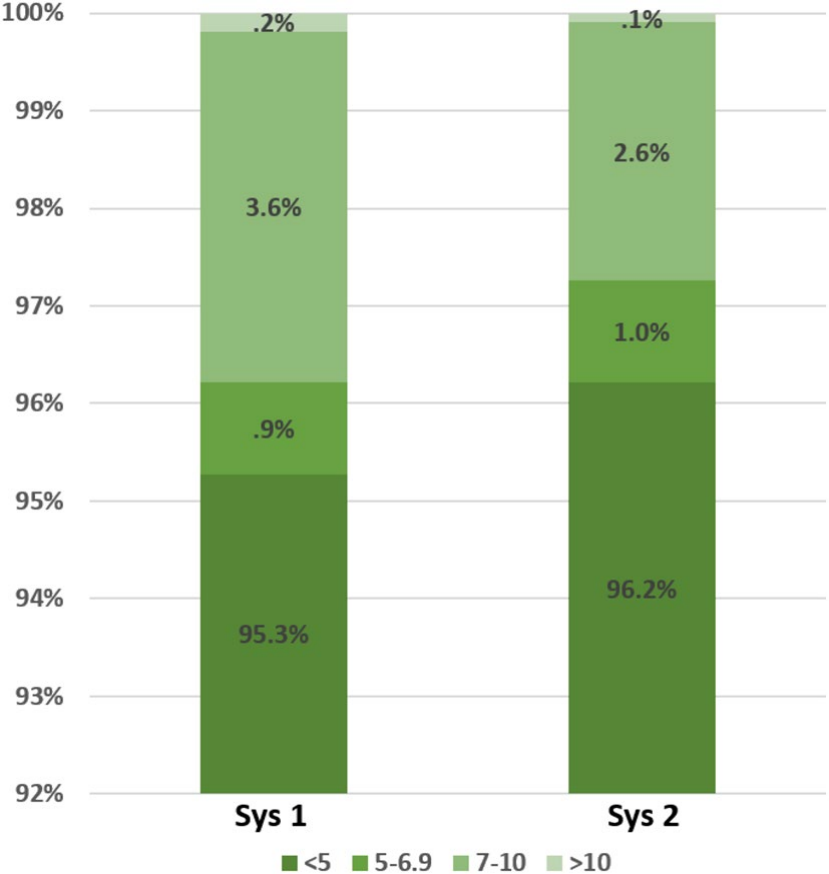
Percentage of measurements with a delta of less than 5, 5–7, 7–10 or above 10 mmHg between the reference device and the BB-613WP systolic measurements. *Sys1* first systolic measurement. *Sys2* second systolic measurement.

The mean deltas for the first (rest) measurement between the reference and the BB-613WP device for systolic and diastolic readings were -0.1 ± 3.6 mmHg and 0 ± 3.5, respectively. The delta for the second (exercise) diastolic measurement was statistically significant but the mean deltas for this second measurement were well below the 5 ± 8 mmHg threshold dictated by the ISO 81060-2:2013 (Table 4). The percentage of systolic measurements with a delta of less than 5, 5–7, 7–10 or above 10 mmHg between the reference and the BB-613WP devices for the first (rest) measurement were: 95.3%, 0.9%, 3.6% and 0.2%, respectively. Similar distribution was demonstrated for the second (exercise) measurement (Fig. 2).

**Table 4:** Evaluation of the Delta between the reference device and the BB-613WP device measurements. N—Number of participants. SD—Standard Deviation.

	First measurement (n = 1057)		Second measurement (n = 491)	
	Systolic	Diastolic	Systolic	Diastolic
Mean	− 0.08	− 0.01	0.26	− 1.14
SD	3.6	3.5	4	4.4
*P* for delta = 0	.49	.98	.17	< .001

### Agreement between the devices

**Figure 3:**
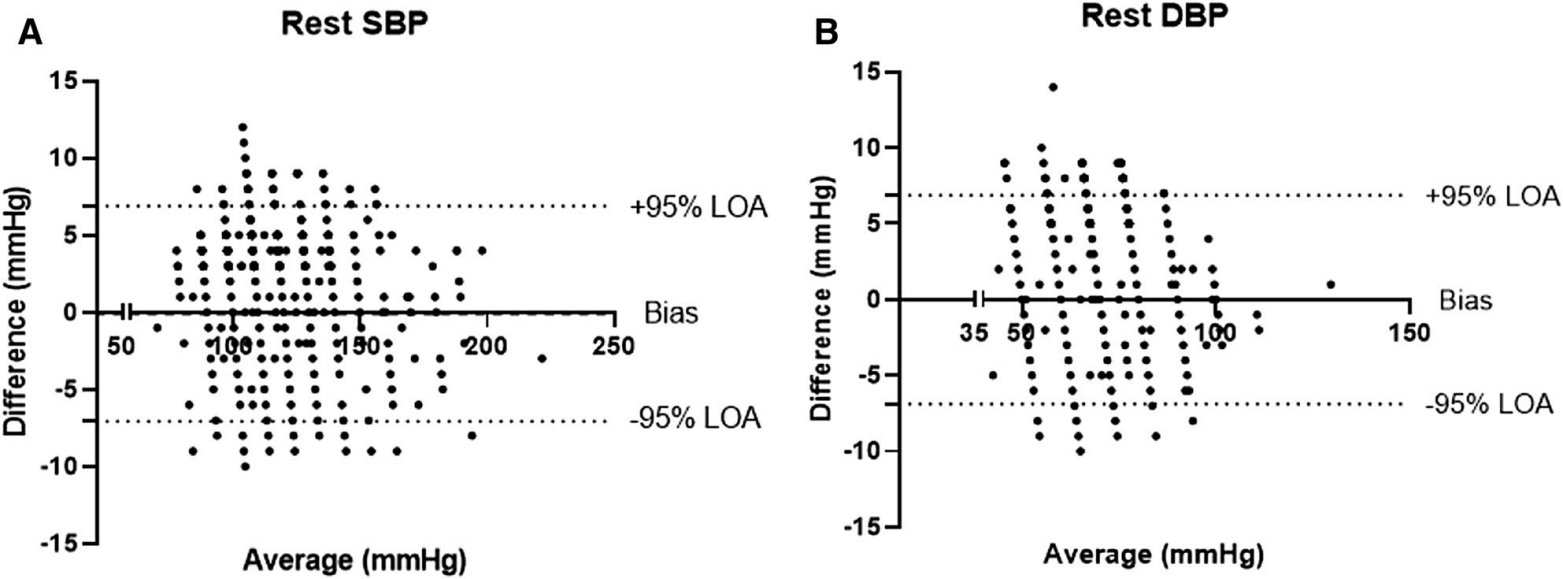
Bland Altman agreement plot of the first blood pressure measurement (n = 1057). (**A**) Systolic blood pressure. (**B**) Diastolic blood pressure.

**Figure 4:**
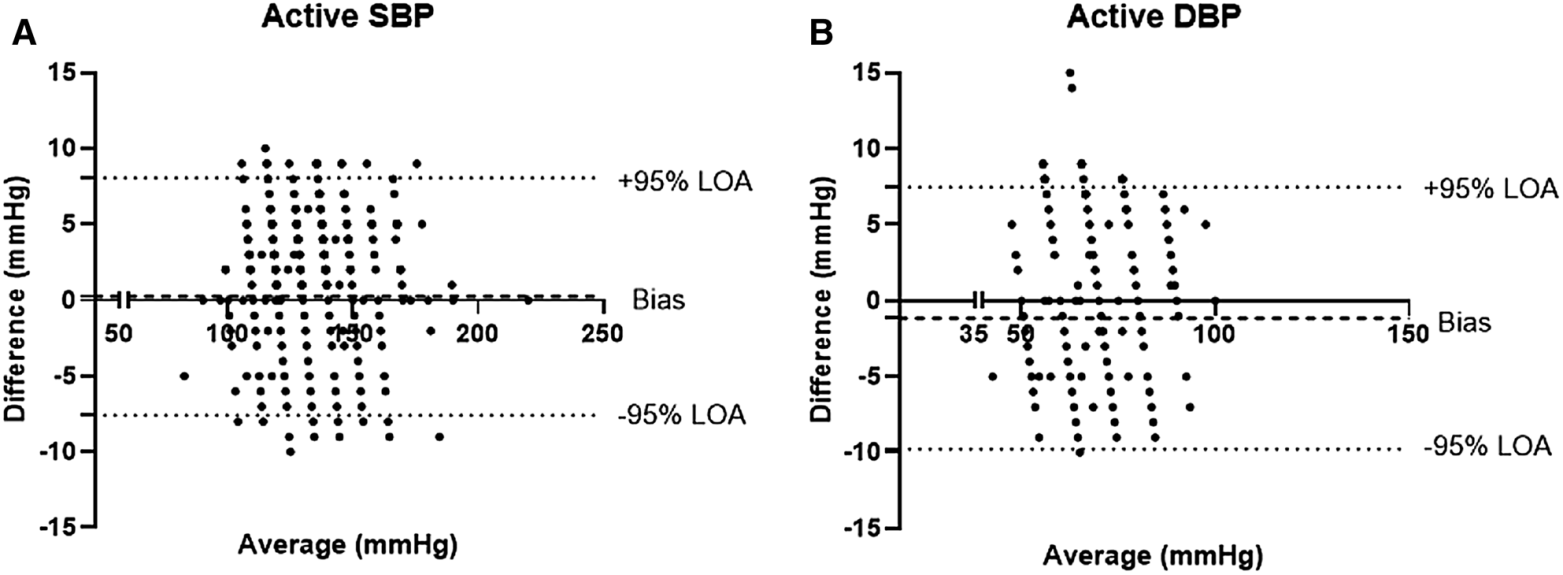
Bland Altman agreement plot of the second blood pressure measurement (n = 491). (**A**) Systolic blood pressure. (**B**) Diastolic blood pressure.

**Figure 5:**
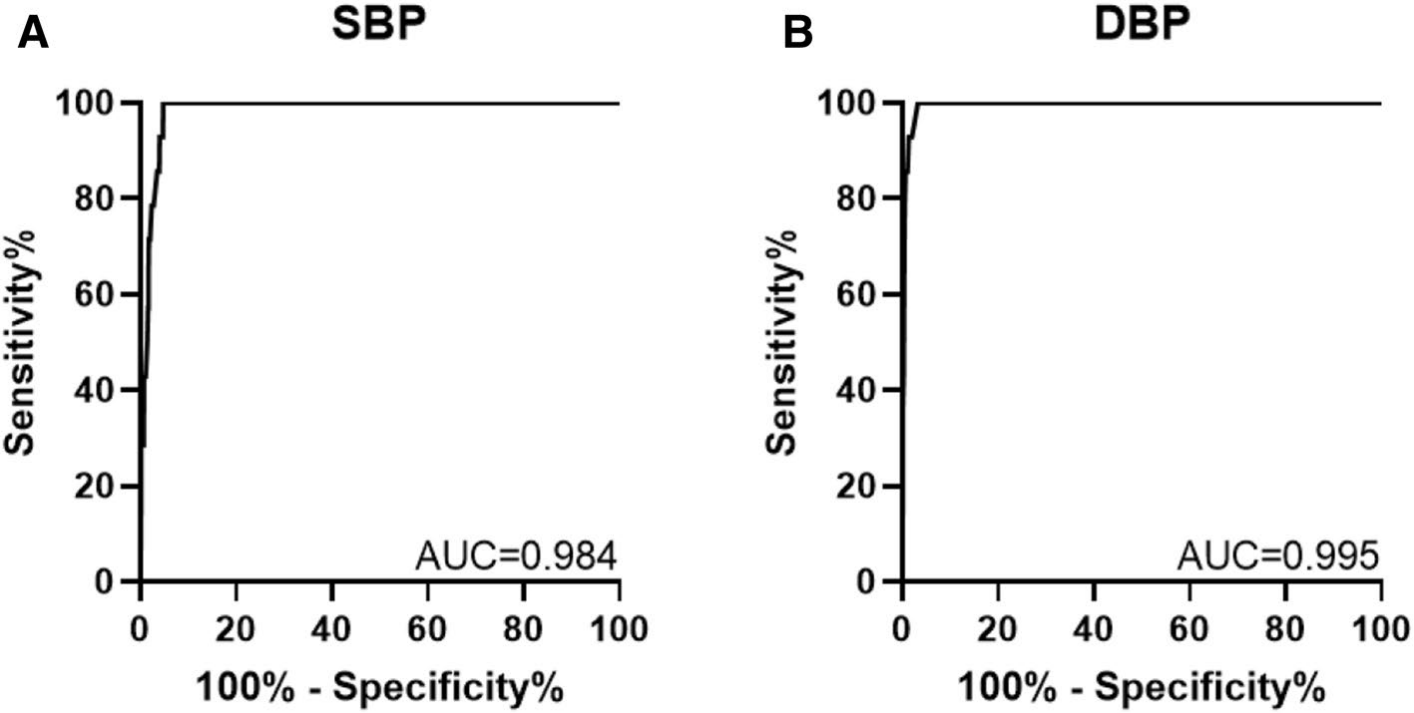
ROC curves analysis illustrating the diagnostic ability of the BB-613WP device in comparison to the standard reference method (sphygmomanometer device).

High agreement between the BB-613WP and the reference devices was demonstrated (Table 5). 100% sensitivity was detected in identifying high and normal blood pressure with specificity of above 92.4%. The Kappa agreement demonstrated substantial agreement between the devices. Bland–Altman agreement plots are presented for SBP and DBP in Figs. 3 and 4, respectively. No systemic difference was detected. Only a small bias was detected for healthy subjects— − 0.08 [− 7.06, 6.90] for systolic BP and 0.002 [− 6.88, 6.87] for diastolic BP. For hypertensive subjects, a slightly higher bias was detected—0.49 [− 7.13, 6.14] for systolic BP and − 0.63 [− 7.11, 5.86] for diastolic BP. The ability to detect and identify patient with hypertension (SBP > 140 mmHg or DBP > 90 mmHg) with the BB-613WP was very similar to the reference device (SBP: AUC = 0.984, DBP: AUC = 0.995) (Fig. 5).

**Table 5:** Percent agreement between the reference device and the BB-613WP in identifying high and normal blood pressure. Hypertension was defined as a systolic value of > 140 mmHg or a diastolic value of > 90 mmHg. Kappa is given as a value (lower–upper limit).

	Sensitivity (%)	Specificity (%)	Agreement (%)	Kappa
Hypertension	100	92.4	94.9	0.89 [0.79–0.98]
No Hypertension	100	96.2	96.5	0.74 [0.66–0.83]

### Sub-group analysis

97 subjects (9.15%) had reported a previous diagnosis of hypertension. They were significantly older and weighed more than the general subject population (66 vs. 32 years, and 74.8 vs. 67.4 kg, respectively). They also had more background diseases such as diabetes, hypercholesterolemia, CVA and COPD. ICC showed high reliability for both the systolic and diastolic measurements in subjects with or without reported hypertension. The average difference and the SD between the reference cuff-based device and the BB-613WP in subjects with and without diagnosis of hypertension were within the required limits, according to the ISO 81060-2:2013 (see supplemental file).

## Discussion

The 2017 guidelines published by The American College of Cardiology/American Heart Association (ACC/AHA) encourage changes in the management of hypertension. They recommend a holistic approach including the use of team-based care, routine use of home and ambulatory blood pressure monitoring (out-of-office BP measurements), and they stress the importance of consistent, accurate, and standardized measurements of BP^13–15^. The change in the guideline’s definitions of hypertension resulted in an abrupt surge in the proportion of hypertensive adults in the United States^17,18^. Moreover, the new target of treatment for BP is accordingly lower.

Known discrepancies between office and home BP values, defined as white-coat hypertension and masked hypertension, together with the narrower BP goals now recommended, require closer BP monitoring and a need to frequent the use of out-of-office BP measurements. Recent estimates are that more than 800 million adults worldwide have an SBP of 140 mmHg or higher. Hypertension’s association with stroke, cardiovascular disease, heart failure, and chronic kidney disease places it with other leading preventable causes of death in the United States, and its consequences are projected to increase^14,15,19^.

Expanding the use of home blood pressure measurements is important yet challenging. The quality of measurements among hypertensive patients varies, and teaching people to perform good-quality and reliable blood pressure measurements necessitates dedicated resources and is challenging, particularly in demanding clinical settings^18^. Ambulatory Blood Pressure (ABP) measurement is now acknowledged as the preferred method for predicting the risk of cardiovascular events relative to an individual’s BP level^20^. Following these perceptions, questions have been raised on the accuracy, and consequently the role, of manual BP measurement in routine clinical practice^14,15,21^ (see also https://www.nice.org.uk/guidance/cg127). Routine office BP measurements (both oscillometric and manual) are not only more susceptible to a “white coat effect”, but are also less accurate, correlate quite poorly with ABP, and are associated with approximating readings to the nearest zero value^22–24^.

Reducing human involvement in the measurement of BP in the office, especially if considering that proper measurement techniques are rarely followed, is suggested to improve the quality of readings^20,23^.

The above-mentioned insights and findings emphasize the importance of having a device that allows better practice and compliance by users by being non-invasive, cuffless, and wireless, enabling multiple measurements without disrupting the user’s daily routine.

The subject population in this study was heterogenous and represented both genders. 26.7% had background chronic diseases (Table 1) including medical conditions and characteristics that increase the risk of cardiovascular diseases, and 16% were taking medications on a chronic basis. 8.6% were smokers. This subject population represents the general Israeli population that visits the local hypertension clinics. 9.1% of the included subjects reported being pre-diagnosed with hypertension, with an average age of 66 years and with an average BMI of normal-high (mildly overweight).

A first blood pressure measurement was recorded in 1057 subjects. The average difference between the cuff-based reference device and the BB-613WP was 0.1 mmHg (SD of 3.6) for systolic BP measurements and 0 mmHg (SD of 3.5) for diastolic BP measurements, i.e. statistically insignificant and below the threshold levels defined by different regulatory protocols such as the ISO 81060-2:2013.

The Intraclass Correlation Coefficient (ICC) presents high reliability of the BB-613WP device with a 0.992 value for systolic BP and a 0.975 value for diastolic BP.

In the 491 subjects for whom a second measurement was taken, the average systolic BP in the reference device increased from 117.1 to 135.0 mmHg, without any change in the average diastolic BP. The BB-613WP device detected these changes with high reliability—ICC = 0.989 for systolic BP and 0.957 for diastolic BP. This means that the novel PPG-based device can detect and follow changes in BP along time and not only when the BP is stable and without any changes. Consistently, the average changes and the SD are well under the required by the ISO 81060-2:2013.

The agreement percentage between the reference device and the BB-613WP in identifying high and normal blood pressure was high (94.85%, κ = 0.881 and 97.19%, κ = 0.783, respectively). In 15.2% of the non-hypertensive subjects the reference cuff-based device showed a high BP value, while the BB-613WP device showed similar high BP values in 92.8% of these subjects. Bland–Altman plots demonstrate that the agreement between the BB-613WP and the reference device is within the limits of the ISO 81060-2:2013 definitions, suggesting good/acceptable agreement in all subjects. A subgroup analysis of subjects with or without reported hypertension demonstrated similar results.

In conclusion, when comparing BP values measured by the standard cuff-based device to the novel BB-613WP device in subjects with both normal and high BP levels, we show that the mean value of the differences in systolic and diastolic measurements for all subjects is less than 5.0 mmHg and the SD is less than 8.0 mmHg, as required by different regulatory protocols such as the ISO 81060-2:2013. This indicates that the PPG-based BB-613WP device can be used as an accurate and reliable BP measurement device.

Beyond its accuracy and reliability, a PPG-based device is simpler to place and use than the cuff-based device, reducing the involvement needed by health personnel. These findings may change the way these parameters will be monitored in the near future, both in pre-hospital and in-hospital settings, as well as in home care scenarios over prolonged periods of time. Further studies should be undertaken to strengthen this data.

This was a short-term study with only one or two measurements collected and being compared between the two devices, minutes after the reference baseline value (calibration) took place in the BB-613WP device. As such, a prolonged protocol with more measurements per subject is needed to substantiate our findings. That said, already in this setting we were able to show the accuracy and the usability of such a wearable, wireless and non-invasive device. We are now in the process of concluding several other clinical studies addressing this limitation.

Based on the ISO 81060-2:2013, measurements of BP should be taken by two investigators, with the reference value being the average of these measurements. This was not performed in the study as we could not interfere and change the way the designated hypertension screening stations operate. This also is being addressed in several other clinical studies using the BB-613WP.

When EMS personnel take measurements with the cuff-based device, they round the numbers to the nearest 5 mmHg value. This has an influence on the comparison and is seen in the Bland–Altman plots. The research team decided not to change this pattern, with the intent to prevent any influence and allow the EMS personnel to continue with their tasks as usual.

When using any sort of physical exercise to increase the BP, it will decay back to normal rapidly upon cessation of the exercise, therefore methods for standardization should take no more than few seconds of measurement. The research team did not want to change and interfere too much with the routine measurement of BP. Thus, despite this challenging environment, subjects that performed the 5 min exercise had the BB-613WP on them during the session, and upon completion of the exercise session BP measurement using the cuff-based device was immediately taken and in parallel the value in the BB-613WP device was recorded, as detailed in the methods.

ISO 81060-2:2013 was replaced by ISO 81060-2:2018. By the time the study was conducted, ISO 81060-2:2013 was still valid. Importantly, ISO 81060-2:2013 is still accepted by regulators.

## Methods

This comparative prospective study was approved by the institutional review board (IRB) of the Tel-Aviv Medical Center, Tel-Aviv, Israel (0032-15-TLV).

### Study population

Male and female volunteers were recruited with no age restrictions. Each came for a BP checkup provided by the Israeli Emergency Medical Services (EMS) in designated BP measurement public screening stations deployed across the country. Each signed an informed consent form as defined by the Institutional Review Board (IRB). Informed consent was provided by the parents of subjects under the age of 18 years. The research team received coded numbers of the participants without any personal identifiers. Recruitment of subjects lasted for a period of two months, aiming to recruit more than the minimal sample size defined by the ISO 81060-2:2013 (85 subjects and 255 valid paired measurements).

### The BB-613WP device

The BB-613WP is a wrist-worn or skin attached device indicated for use in measuring and displaying functional oxygen saturation of arterial hemoglobin (%SpO2) and pulse rate using photoplethysmography (PPG) technology. Most commercially available devices transmit light in specific red (~ 650 nm) and infrared (~ 880 nm) wavelengths through the tested tissue, and a detector on the other side absorbs the transmitted light. These wavelengths have a unique absorbance pattern upon interaction with oxy- and deoxyhemoglobin. The detector measures the changing absorbance at each of the wavelengths allowing it to determine the absorbance resulting from the pulsating arterial blood alone excluding venous blood, skin, bone, muscle, and fat. The BB-613WP uses a unique reflective PPG technology, where, unlike most devices, the light source (Light Emitting Diodes, LEDs) and sensor array are placed on the same side (backside) of the device. As the LEDs transmit light into the subject’s skin, part of this light is reflected from the tissue and is detected by a photodiode detector. The high temporal and quantitative resolution of the device allows it to capture minute changes in tissue reflectance, calculating numerous vital signs derived from pulse contours, including advanced hemodynamic parameters such as tracking changes in blood pressure. This is based on Pulse Wave Transit Time (PWTT) which is obtained utilizing pulse measurements from the integrated SpO2 sensor, following a calibration process using an oscillometric blood pressure monitor. Once a calibration measurement is taken using the cuff-based BP device, the values are entered into a user’s application. The calibration is valid for three months, after which a new calibration measurement should be taken and introduced into the application using the same method.

The results are displayed on the LCD screen of the wristwatch device as well as on the mobile application installed on the user’s smartphone. From there, the data is transmitted in real-time to a web application used by health care providers.

### Study protocol

Comparison between the devices was performed based on the International Organization for Standardization (ISO) guideline for non-invasive sphygmomanometers (ISO 81060-2:2013^25^), which is considered by the FDA as a relevant protocol for regulatory use.

**Figure 6:**
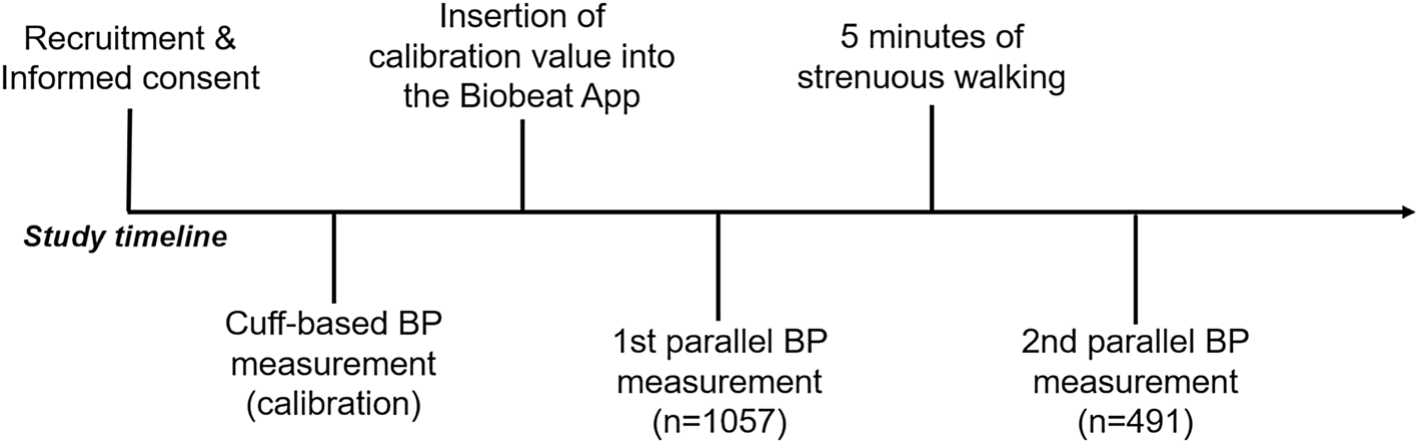
Description of the different stages of the study.

BP measurements were taken by skilled EMS personnel. In each volunteer, a reference BP measurement using a cuff-based device (Welch Allyn DuraShock DS65 hand sphygmomanometer, Skaneateles Falls, NY 13153, USA) was taken and used as the baseline calibration value for the PPG-based wearable device (Fig. 6). Next, the wearable device was attached to each of the subjects by the EMS personnel and left for 30 s to acquire a reading of the PPG signal while the volunteers were sitting. This was repeated on the other hand to show there are no differences. After obtaining a signal, BP was measured concomitantly in all subjects using both devices—the standard manometry device mentioned earlier on one hand and the BB-613WP on the opposite arm. Since the manometry device occludes blood flow to the arm, PPG measurements cannot be obtained if the device is located on that same arm. When comparing the BB-613WP to a cuff-based BP device, and since it takes time between the systolic measurement and the diastolic measurement using the cuff-based device, the systolic value of the BB-613WP is recorded once systolic value is reached in the cuff-based device, and once the diastolic value is reached in the cuff-based device, the diastolic measurement in the BB-613WP device is recorded.

Healthy subjects with no known background diseases were asked whether they would agree to perform a short session of exercise (5 min of walking fast near the measurement site). Those who agreed and signed were included in this sub-group. The aim was to change the BP and to show that the BB-613WP can detect this change between the two measurements. Adverse events were recorded by the research team, such as local wrist irritation (where the wristwatch was placed), or any other complaint by the user.

### Statistical analysis

According to ISO 81060-2:2013 the PPG device can be considered as equal to the reference when the mean value of the differences in paired systolic and diastolic readings between the reference and the PPG device is equal to or less than 5 mmHg and the Standard Deviation (SD) is no greater than 8 mmHg. The PPG device reliability was determined by the Interclass Correlation Coefficient (ICC) in which an ICC ≥ 0.9 indicates “excellent reliability”^26^. Correlation analysis was also performed using Pearson correlation, and agreement was evaluated based on the Bland–Altman method using 95% limits of agreement (LOA). The agreement in the classification (detection) of hypertensive cases (defined by the European Hypertension Society to be > 140 mmHg systolic pressure or > 90 mmHg diastolic pressure) was evaluated using Kappa agreement. Hypertension (SBP > 140 mmHg or DBP > 90 mmHg) detection ability was evaluated using ROC curves analysis. Pre-specified subgroup analysis of patients with and without previously diagnosed HTN was also performed. P values were set at 0.05. Data analysis was performed using the IBM SPSS Statistics for Windows, Version 23.0, IBM Corp (Armonk, NY), released 2015 and GraphPad Prism, version 8.0.2 (San Diego, CA) released 2019.

### Ethical approval

All procedures performed in this clinical study were in accordance with the ethical standards of the local IRB (Approval Number 0032-15-TLV) and with the 1964 Helsinki declaration and its later amendments or comparable ethical standards.

### Informed consent

Informed consent was obtained from all individual participants included in the study. Informed consent was provided by the parents of subjects under the age of 18 years.

## Supplementary information

Supplementary Information.

## Abbreviations

AAMI: Association for the Advancement of Medical Instrumentation
ABP: Ambulatory blood pressure
ACC: American College of Cardiology
AHA: American Heart Association
AOBP: Automated office blood pressure
BP: Blood pressure
DBP: Diastolic blood pressure
EMS: Emergency Medical Services
ICC: Intraclass correlation coefficient
IRB: Institutional Review Board
ISO: International Organization for Standardization
MAP: Mean arterial pressure
PPG: Photoplethysmography
SBP: Systolic blood pressure
SD: Standard deviation
