# Supplementary material for: Comparing blood pressure measurements between a photoplethysmography-based and a standard cuff-based manometry device
Source: Sci Rep. 2020 Sep 30;10:16116. doi: 10.1038/s41598-020-73172-3 (PMC7527983; doi:10.1038/s41598-020-73172-3)
Supplement: Supplementary file 1 — Supplementary Information. [file 41598_2020_73172_MOESM1_ESM.docx]

**Supplemental file**

**Comparing Blood Pressure Measurements Between a Photoplethysmography-Based** **and a Standard Cuff-Based Manometry Device**

Dean Nachman, MD^1,2^, Yftach Gepner, PHD^3^, Nir Goldstein, PHD^3^, Eli Kabakov, MD, MPH^1^, Arik Ben Ishay^4^, Romi Littman^4^, Yuval Azmon, MD^5^, Eli Jaffe, PhD^6^, Arik Eisenkraft, MD, MHA^1,4*^

*^1^The Institute for Research in Military Medicine, The Hebrew University Faculty of Medicine, Jerusalem, and the Israel Defense Force Medical Corps, Jerusalem, Israel; ^2^Heart Institute,, Hadassah Ein Kerem Medical Center, Jerusalem, Israel;* ^3^*Department of Epidemiology and Preventive Medicine, School of Public Health, Sackler Faculty of Medicine, and Sylvan Adams Sports Institute, Tel-Aviv University, Tel-Aviv, Israel ^4^Biobeat Technologies LTD, Petah Tikva, Israel; ^5^Obstetrics and Gynecology department, Hillel Yaffe Medical Center and The Rappaport Faculty of Medicine, The Technion, Israel; ^6^Magen David Adom, Israel National Emergency Medical Services, Kiryat Ono, Israel*

Correspondence should be addressed to Dr. Arik Eisenkraft, MD, MHA; Institute for Research in Military Medicine, Faculty of Medicine, The Hebrew University of Jerusalem and the Israel Defense Force Medical Corps, POB 12272, Jerusalem 91120, Israel. Tel: +972-52-9210896, +972-2-6757657, Fax: +972-2-6757660, email: aizenkra@gmail.com

**
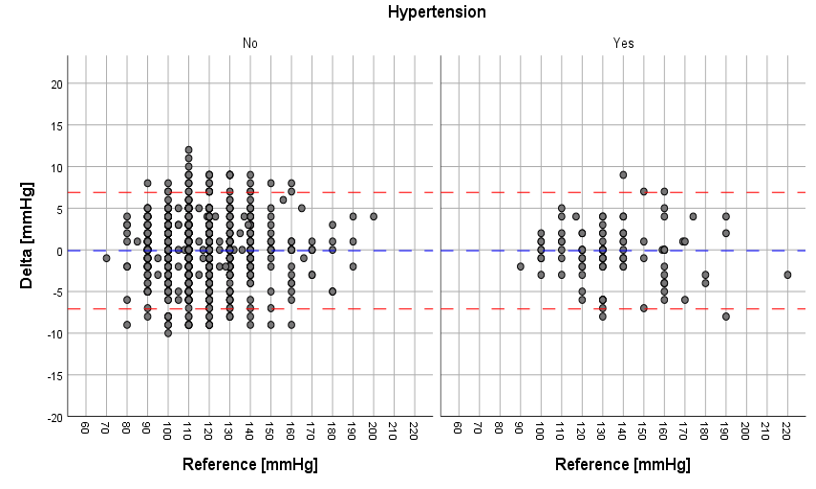
**

**Fig 7 (supplement)** Bland Altman agreement plot of the first systolic blood pressure measurement in subjects with and without reported hypertension (n=97 and 960, respectively).

**
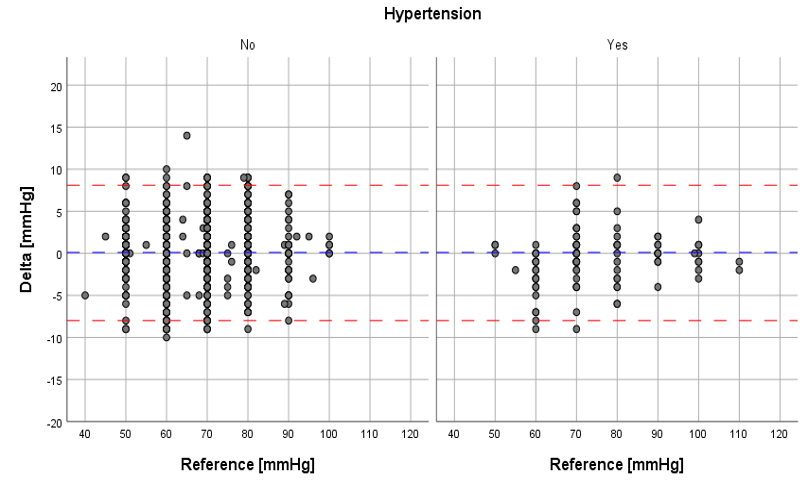
**

**Fig 8 (supplement)** Bland Altman agreement plot of the first diastolic blood pressure measurement in subjects with and without reported hypertension (n=97 and 960, respectively).

**Table 6 (supplemental).** First BP values measured by both devices in subjects with and without reported hypertension.

|  |  | | No | Yes | P-value |
| --- | --- | --- | --- | --- | --- |
| Reference Systolic | | N | 960 | 97 | <.001 |
| (mmHg) | | Mean ± SD | 115.2±17.9 | 136.1±24.1 |  |
|  | | Minimum | 70.0 | 90.0 |  |
|  | | Maximum | 200.0 | 220.0 |  |
| Reference Diastolic | | N | 960 | 97 | <.001 |
| (mmHg) | | Mean ± SD | 67.6±10.6 | 74.9±13.8 |  |
|  | | Minimum | 40.0 | 50.0 |  |
|  | | Maximum | 100.0 | 110.0 |  |
| BB-613PW Systolic | | N | 960 | 97 | <.001 |
| (mmHg) | | Mean ± SD | 115.2±18.2 | 136.6±24.6 |  |
|  | | Minimum | 71.0 | 92.0 |  |
|  | | Maximum | 196.0 | 223.0 |  |
| BB-613PW Diastolic | | N | 960 | 97 | <.001 |
| (mmHg) | | Mean ± SD | 67.5±11.0 | 75.5±13.7 |  |
|  | | Minimum | 41.0 | 49.0 |  |
|  | | Maximum | 100.0 | 112.0 |  |

SD - Standard deviation. N - number of participants.

**Table 7 (supplemental).** Interclass Correlation Coefficient for the first and second blood pressure measurements of subjects with and without reported hypertension.

|  | | | Hypertension | | | | No Hypertension | | | |
| --- | --- | --- | --- | --- | --- | --- | --- | --- | --- | --- |
|  |  |  | Sys1 | Sys2 | Dias1 | Dias2 | Sys1 | Sys2 | Dias1 | Dias2 |
| Intraclass Correlation Coefficient | Value | | 0.995 | NA | 0.985 | NA | 0.990 | 0.988 | 0.973 | 0.956 |
|  | 95%CI | Upper limit | 0.993 | NA | 0.977 | NA | 0.989 | 0.986 | 0.969 | 0.944 |
|  |  | Lower Limit | 0.997 | NA | 0.990 | NA | 0.991 | 0.990 | 0.976 | 0.965 |
|  | Sig | | <.001 | NA | <.001 | NA | <.001 | <.001 | <.001 | <.001 |

NA= not applicable, as there were only 2 subjects in this group. Sys1 - first systolic measurement; Sys2 - second systolic measurement; Dias1 - first diastolic measurement; Dias2 - second diastolic measurement.

**Table 8 (supplemental).** Evaluation of the mean difference in the first measurement between the reference device and the BB-613PW device in subjects with and without reported hypertension.

|  | Hypertension | | No Hypertension | |
| --- | --- | --- | --- | --- |
|  | Systolic | Diastolic | Systolic | Diastolic |
| N | 97 | 97 | 961 | 960 |
| Mean | -0.5 | -0.6 | 0.0 | 0.1 |
| SD | 3.4 | 3.3 | 3.6 | 3.5 |
| P for delta =0 | .172 | .048 | .774 | .545 |

N - Number of participants. SD - Standard deviation
